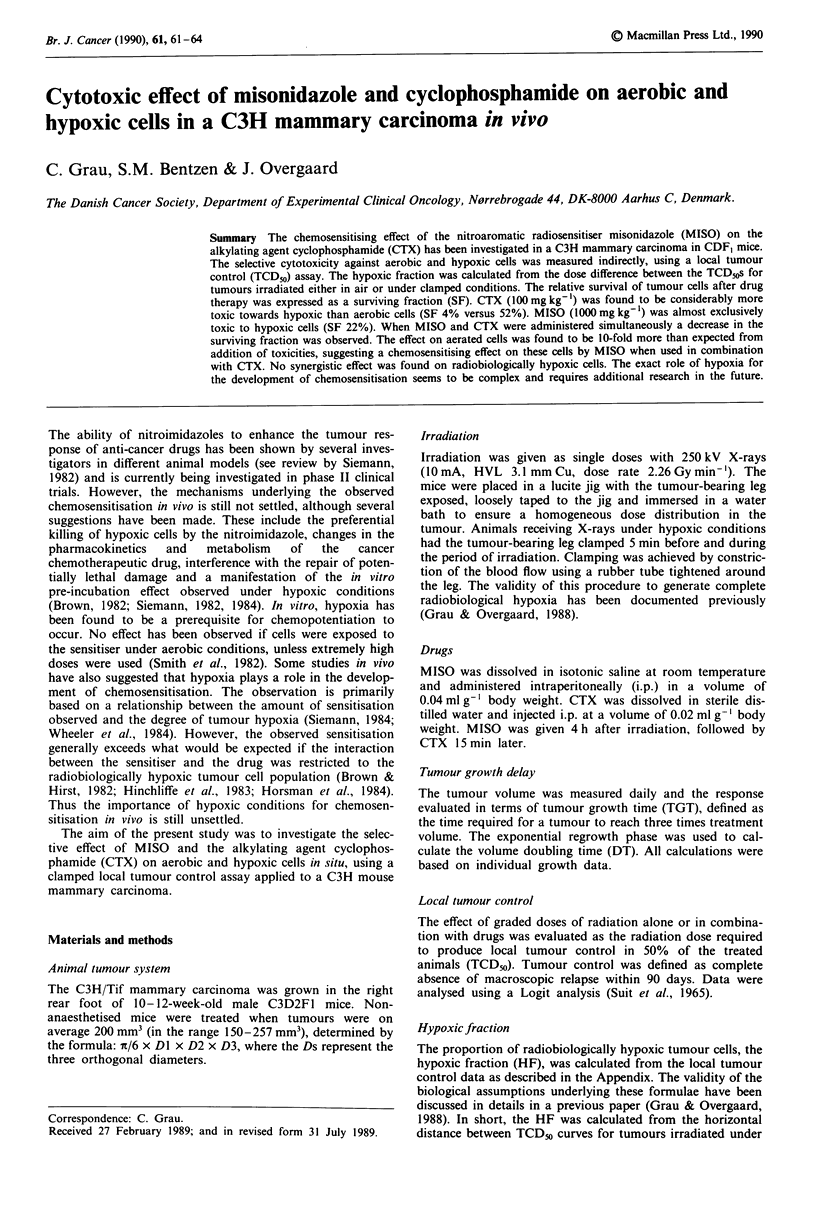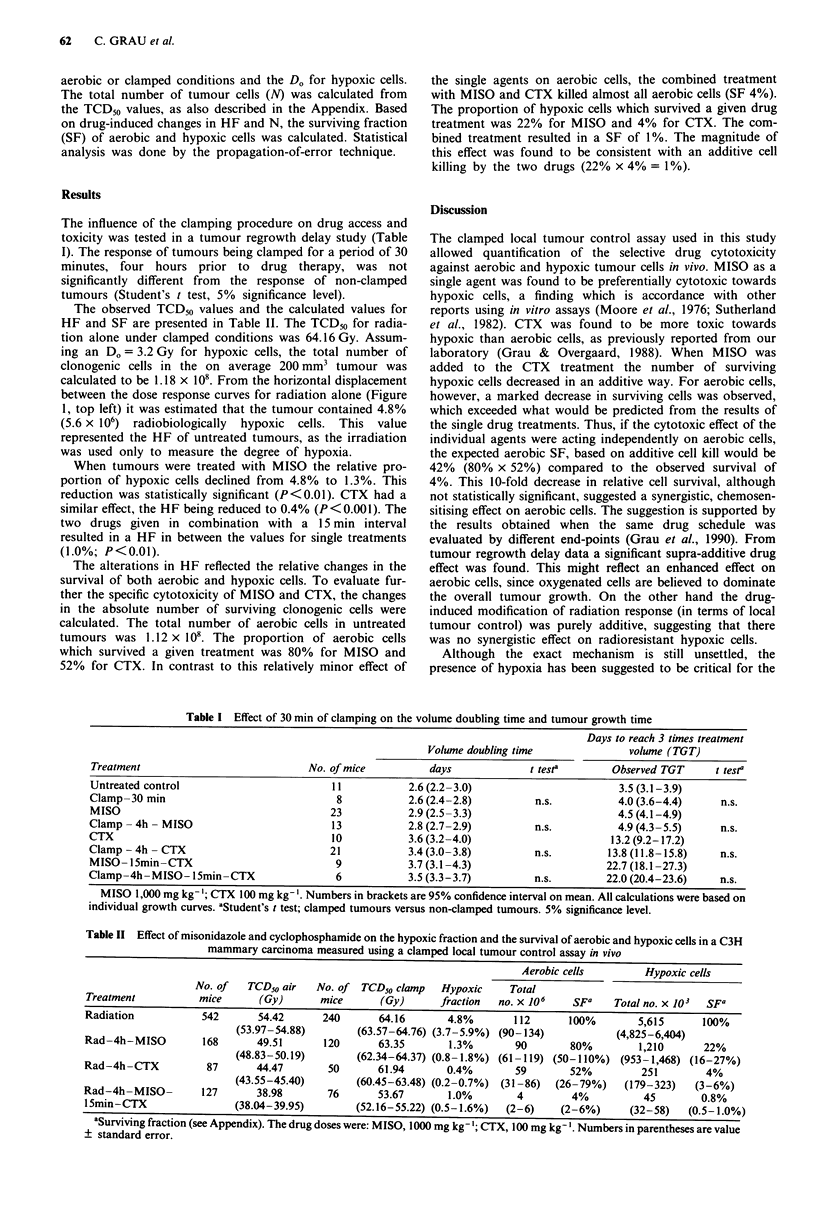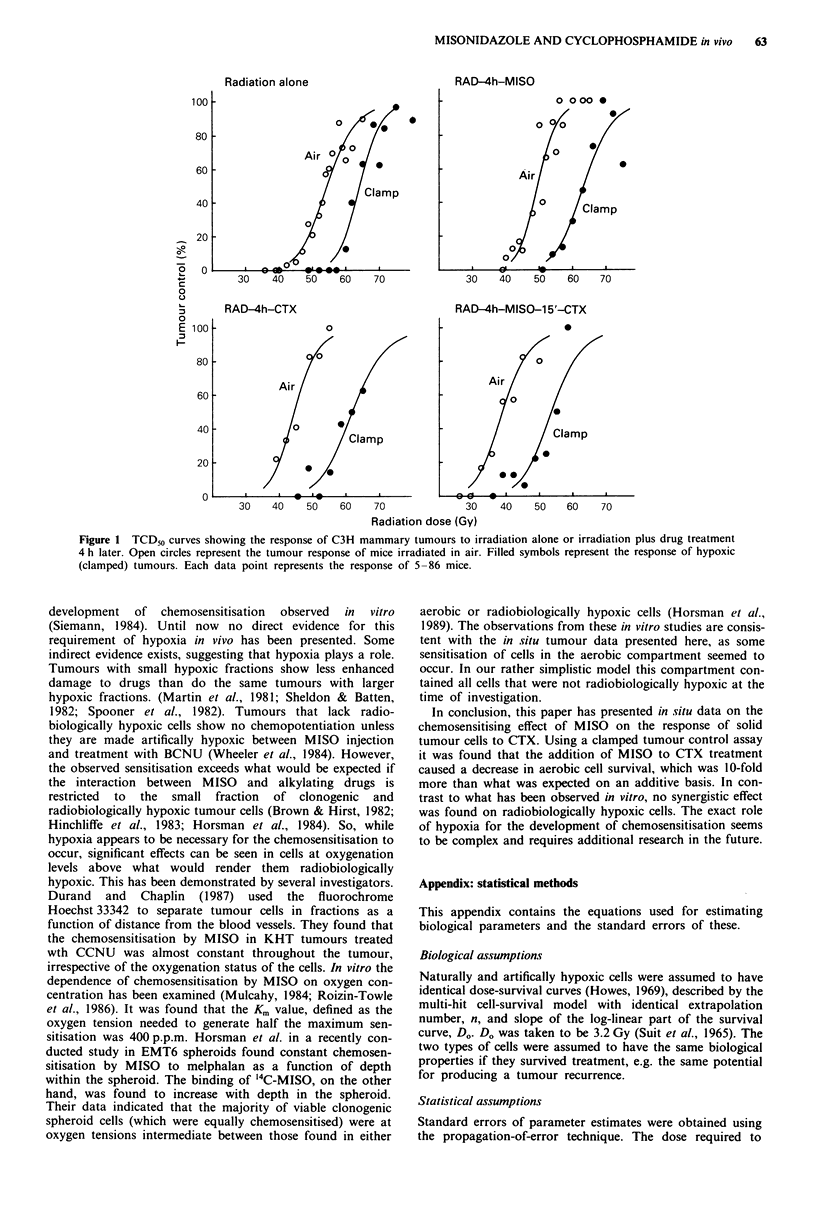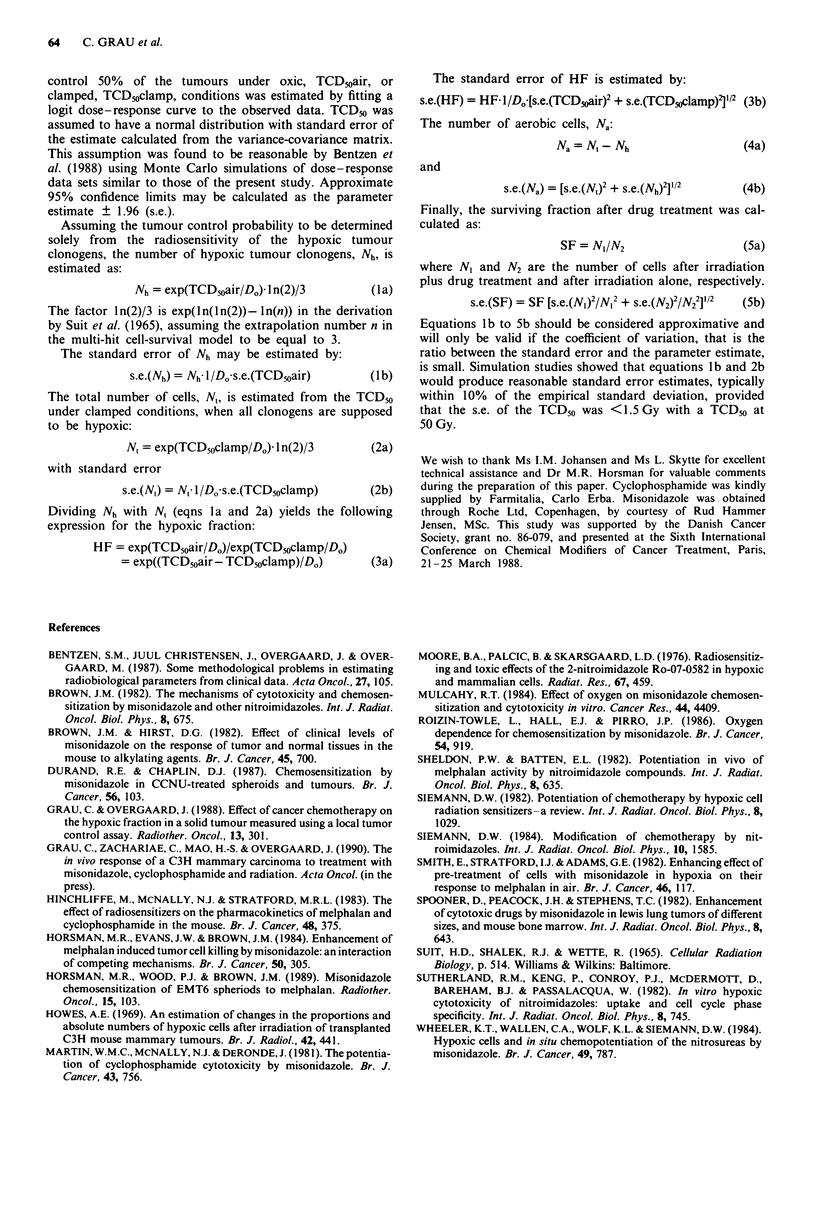# Cytotoxic effect of misonidazole and cyclophosphamide on aerobic and hypoxic cells in a C3H mammary carcinoma in vivo.

**DOI:** 10.1038/bjc.1990.13

**Published:** 1990-01

**Authors:** C. Grau, S. M. Bentzen, J. Overgaard

**Affiliations:** Danish Cancer Society, Department of Experimental Clinical Oncology, Aarhus.

## Abstract

The chemosensitising effect of the nitroaromatic radiosensitiser misonidazole (MISO) on the alkylating agent cyclophosphamide (CTX) has been investigated in a C3H mammary carcinoma in CDF1 mice. The selective cytotoxicity against aerobic and hypoxic cells was measured indirectly, using a local tumour control (TCD50) assay. The hypoxic fraction was calculated from the dose difference between the TCD50S for tumours irradiated either in air or under clamped conditions. The relative survival of tumour cells after drug therapy was expressed as a surviving fraction (SF). CTX (100 mg kg-1) was found to be considerably more toxic towards hypoxic than aerobic cells (SF 4% versus 52%). MISO (1000 mg kg-1) was almost exclusively toxic to hypoxic cells (SF 22%). When MISO and CTX were administered simultaneously a decrease in the surviving fraction was observed. The effect on aerated cells was found to be 10-fold more than expected from addition of toxicities, suggesting a chemosensitising effect on these cells by MISO when used in combination with CTX. No synergistic effect was found on radiobiologically hypoxic cells. The exact role of hypoxia for the development of chemosensitisation seems to be complex and requires additional research in the future.


Br. J. Cancer (1990), 61, 61–64 © Macmillan Press Ltd., 1990

### Cytotoxic effect of misonidazole and cyclophosphamide on aerobic and hypoxic cells in a C3H mammary carcinoma *in vivo*

C. Grau, S.M. Bentzen & J. Overgaard

*The Danish Cancer Society, Department of Experimental Clinical Oncology, Nørrebrogade 44, DK-8000 Aarhus C, Denmark.*

**Summary** The chemosensitising effect of the nitroaromatic radiosensitiser misonidazole (MISO) on the alkylating agent cyclophosphamide (CTX) has been investigated in a C3H mammary carcinoma in $CDF_1$ mice. The selective cytotoxicity against aerobic and hypoxic cells was measured indirectly, using a local tumour control ($TCD_{50}$) assay. The hypoxic fraction was calculated from the dose difference between the $TCD_{50}$s for tumours irradiated either in air or under clamped conditions. The relative survival of tumour cells after drug therapy was expressed as a surviving fraction (SF). CTX (100 mg $kg^{-1}$) was found to be considerably more toxic towards hypoxic than aerobic cells (SF 4% versus 52%). MISO (1000 mg $kg^{-1}$) was almost exclusively toxic to hypoxic cells (SF 22%). When MISO and CTX were administered simultaneously a decrease in the surviving fraction was observed. The effect on aerated cells was found to be 10-fold more than expected from addition of toxicities, suggesting a chemosensitising effect on these cells by MISO when used in combination with CTX. No synergistic effect was found on radiobiologically hypoxic cells. The exact role of hypoxia for the development of chemosensitisation seems to be complex and requires additional research in the future.

The ability of nitroimidazoles to enhance the tumour response of anti-cancer drugs has been shown by several investigators in different animal models (see review by Siemann, 1982) and is currently being investigated in phase II clinical trials. However, the mechanisms underlying the observed chemosensitisation *in vivo* is still not settled, although several suggestions have been made. These include the preferential killing of hypoxic cells by the nitroimidazole, changes in the pharmacokinetics and metabolism of the cancer chemotherapeutic drug, interference with the repair of potentially lethal damage and a manifestation of the *in vitro* pre-incubation effect observed under hypoxic conditions (Brown, 1982; Siemann, 1982, 1984). *In vitro*, hypoxia has been found to be a prerequisite for chemopotentiation to occur. No effect has been observed if cells were exposed to the sensitiser under aerobic conditions, unless extremely high doses were used (Smith *et al.*, 1982). Some studies *in vivo* have also suggested that hypoxia plays a role in the development of chemosensitisation. The observation is primarily based on a relationship between the amount of sensitisation observed and the degree of tumour hypoxia (Siemann, 1984; Wheeler *et al.*, 1984). However, the observed sensitisation generally exceeds what would be expected if the interaction between the sensitiser and the drug was restricted to the radiobiologically hypoxic tumour cell population (Brown & Hirst, 1982; Hinchliffe *et al.*, 1983; Horsman *et al.*, 1984). Thus the importance of hypoxic conditions for chemosensitisation *in vivo* is still unsettled.

The aim of the present study was to investigate the selective effect of MISO and the alkylating agent cyclophosphamide (CTX) on aerobic and hypoxic cells *in situ*, using a clamped local tumour control assay applied to a C3H mouse mammary carcinoma.

### Materials and methods

### *Animal tumour system*

The C3H/Tif mammary carcinoma was grown in the right rear foot of 10–12-week-old male C3D2F1 mice. Non-anaesthetised mice were treated when tumours were on average 200 $mm^3$ (in the range 150–257 $mm^3$), determined by the formula: $\pi/6 \times D1 \times D2 \times D3$, where the *D*s represent the three orthogonal diameters.

Correspondence: C. Grau.
Received 27 February 1989; and in revised form 31 July 1989.

### *Irradiation*

Irradiation was given as single doses with 250 kV X-rays (10 mA, HVL 3.1 mm Cu, dose rate 2.26 Gy $min^{-1}$). The mice were placed in a lucite jig with the tumour-bearing leg exposed, loosely taped to the jig and immersed in a water bath to ensure a homogeneous dose distribution in the tumour. Animals receiving X-rays under hypoxic conditions had the tumour-bearing leg clamped 5 min before and during the period of irradiation. Clamping was achieved by constriction of the blood flow using a rubber tube tightened around the leg. The validity of this procedure to generate complete radiobiological hypoxia has been documented previously (Grau & Overgaard, 1988).

### *Drugs*

MISO was dissolved in isotonic saline at room temperature and administered intraperitoneally (i.p.) in a volume of 0.04 ml $g^{-1}$ body weight. CTX was dissolved in sterile distilled water and injected i.p. at a volume of 0.02 ml $g^{-1}$ body weight. MISO was given 4 h after irradiation, followed by CTX 15 min later.

### *Tumour growth delay*

The tumour volume was measured daily and the response evaluated in terms of tumour growth time (TGT), defined as the time required for a tumour to reach three times treatment volume. The exponential regrowth phase was used to calculate the volume doubling time (DT). All calculations were based on individual growth data.

### *Local tumour control*

The effect of graded doses of radiation alone or in combination with drugs was evaluated as the radiation dose required to produce local tumour control in 50% of the treated animals ($TCD_{50}$). Tumour control was defined as complete absence of macroscopic relapse within 90 days. Data were analysed using a Logit analysis (Suit *et al.*, 1965).

### *Hypoxic fraction*

The proportion of radiobiologically hypoxic tumour cells, the hypoxic fraction (HF), was calculated from the local tumour control data as described in the Appendix. The validity of the biological assumptions underlying these formulae have been discussed in details in a previous paper (Grau & Overgaard, 1988). In short, the HF was calculated from the horizontal distance between $TCD_{50}$ curves for tumours irradiated under aerobic or clamped conditions and the $D_0$ for hypoxic cells. The total number of tumour cells (*N*) was calculated from the $TCD_{50}$ values, as also described in the Appendix. Based on drug-induced changes in HF and N, the surviving fraction (SF) of aerobic and hypoxic cells was calculated. Statistical analysis was done by the propagation-of-error technique.

### Results

The influence of the clamping procedure on drug access and toxicity was tested in a tumour regrowth delay study (Table I). The response of tumours being clamped for a period of 30 minutes, four hours prior to drug therapy, was not significantly different from the response of non-clamped tumours (Student's *t* test, 5% significance level).

The observed $TCD_{50}$ values and the calculated values for HF and SF are presented in Table II. The $TCD_{50}$ for radiation alone under clamped conditions was 64.16 Gy. Assuming an $D_0 = 3.2$ Gy for hypoxic cells, the total number of clonogenic cells in the on average 200 $mm^3$ tumour was calculated to be $1.18 \times 10^8$. From the horizontal displacement between the dose response curves for radiation alone (Figure 1, top left) it was estimated that the tumour contained 4.8% ($5.6 \times 10^6$) radiobiologically hypoxic cells. This value represented the HF of untreated tumours, as the irradiation was used only to measure the degree of hypoxia.

When tumours were treated with MISO the relative proportion of hypoxic cells declined from 4.8% to 1.3%. This reduction was statistically significant ($P < 0.01$). CTX had a similar effect, the HF being reduced to 0.4% ($P < 0.001$). The two drugs given in combination with a 15 min interval resulted in a HF in between the values for single treatments (1.0%; $P < 0.01$).

The alterations in HF reflected the relative changes in the survival of both aerobic and hypoxic cells. To evaluate further the specific cytotoxicity of MISO and CTX, the changes in the absolute number of surviving clonogenic cells were calculated. The total number of aerobic cells in untreated tumours was $1.12 \times 10^8$. The proportion of aerobic cells which survived a given treatment was 80% for MISO and 52% for CTX. In contrast to this relatively minor effect of the single agents on aerobic cells, the combined treatment with MISO and CTX killed almost all aerobic cells (SF 4%). The proportion of hypoxic cells which survived a given drug treatment was 22% for MISO and 4% for CTX. The combined treatment resulted in a SF of 1%. The magnitude of this effect was found to be consistent with an additive cell killing by the two drugs (22% × 4% = 1%).

### Discussion

The clamped local tumour control assay used in this study allowed quantification of the selective drug cytotoxicity against aerobic and hypoxic tumour cells *in vivo*. MISO as a single agent was found to be preferentially cytotoxic towards hypoxic cells, a finding which is accordance with other reports using *in vitro* assays (Moore *et al.*, 1976; Sutherland *et al.*, 1982). CTX was found to be more toxic towards hypoxic than aerobic cells, as previously reported from our laboratory (Grau & Overgaard, 1988). When MISO was added to the CTX treatment the number of surviving hypoxic cells decreased in an additive way. For aerobic cells, however, a marked decrease in surviving cells was observed, which exceeded what would be predicted from the results of the single drug treatments. Thus, if the cytotoxic effect of the individual agents were acting independently on aerobic cells, the expected aerobic SF, based on additive cell kill would be 42% (80% × 52%) compared to the observed survival of 4%. This 10-fold decrease in relative cell survival, although not statistically significant, suggested a synergistic, chemosensitising effect on aerobic cells. The suggestion is supported by the results obtained when the same drug schedule was evaluated by different end-points (Grau *et al.*, 1990). From tumour regrowth delay data a significant supra-additive drug effect was found. This might reflect an enhanced effect on aerobic cells, since oxygenated cells are believed to dominate the overall tumour growth. On the other hand the drug-induced modification of radiation response (in terms of local tumour control) was purely additive, suggesting that there was no synergistic effect on radioresistant hypoxic cells.

Although the exact mechanism is still unsettled, the presence of hypoxia has been suggested to be critical for the

**Table I** Effect of 30 min of clamping on the volume doubling time and tumour growth time

| *Treatment* | *No. of mice* | *Volume doubling time* days | *t test*[a] | *Days to reach 3 times treatment volume (TGT)* Observed TGT | *t test*[a] |
|---|---|---|---|---|---|
| Untreated control | 11 | 2.6 (2.2–3.0) | | 3.5 (3.1–3.9) | |
| Clamp–30 min | 8 | 2.6 (2.4–2.8) | n.s. | 4.0 (3.6–4.4) | n.s. |
| MISO | 23 | 2.9 (2.5–3.3) | | 4.5 (4.1–4.9) | |
| Clamp - 4h - MISO | 13 | 2.8 (2.7–2.9) | n.s. | 4.9 (4.3–5.5) | n.s. |
| CTX | 10 | 3.6 (3.2–4.0) | | 13.2 (9.2–17.2) | |
| Clamp - 4h - CTX | 21 | 3.4 (3.0–3.8) | n.s. | 13.8 (11.8–15.8) | n.s. |
| MISO–15min–CTX | 9 | 3.7 (3.1–4.3) | | 22.7 (18.1–27.3) | |
| Clamp–4h–MISO–15min–CTX | 6 | 3.5 (3.3–3.7) | n.s. | 22.0 (20.4–23.6) | n.s. |

MISO 1,000 mg $kg^{-1}$; CTX 100 mg $kg^{-1}$. Numbers in brackets are 95% confidence interval on mean. All calculations were based on individual growth curves. [a]Student's *t* test; clamped tumours versus non-clamped tumours. 5% significance level.

**Table II** Effect of misonidazole and cyclophosphamide on the hypoxic fraction and the survival of aerobic and hypoxic cells in a C3H mammary carcinoma measured using a clamped local tumour control assay *in vivo*

| *Treatment* | *No. of mice* | *$TCD_{50}$ air (Gy)* | *No. of mice* | *$TCD_{50}$ clamp (Gy)* | *Hypoxic fraction* | *Aerobic cells* Total no. × $10^6$ | *Aerobic cells* SF[a] | *Hypoxic cells* Total no. × $10^3$ | *Hypoxic cells* SF[a] |
|---|---|---|---|---|---|---|---|---|---|
| Radiation | 542 | 54.42 (53.97–54.88) | 240 | 64.16 (63.57–64.76) | 4.8% (3.7–5.9%) | 112 (90–134) | 100% | 5,615 (4,825–6,404) | 100% |
| Rad–4h–MISO | 168 | 49.51 (48.83–50.19) | 120 | 63.35 (62.34–64.37) | 1.3% (0.8–1.8%) | 90 (61–119) | 80% (50–110%) | 1,210 (953–1,468) | 22% (16–27%) |
| Rad–4h–CTX | 87 | 44.47 (43.55–45.40) | 50 | 61.94 (60.45–63.48) | 0.4% (0.2–0.7%) | 59 (31–86) | 52% (26–79%) | 251 (179–323) | 4% (3–6%) |
| Rad–4h–MISO–15min–CTX | 127 | 38.98 (38.04–39.95) | 76 | 53.67 (52.16–55.22) | 1.0% (0.5–1.6%) | 4 (2–6) | 4% (2–6%) | 45 (32–58) | 0.8% (0.5–1.0%) |

[a]Surviving fraction (see Appendix). The drug doses were: MISO, 1000 mg $kg^{-1}$; CTX, 100 mg $kg^{-1}$. Numbers in parentheses are value ± standard error.

**Figure 1** $TCD_{50}$ curves showing the response of C3H mammary tumours to irradiation alone or irradiation plus drug treatment 4 h later. Open circles represent the tumour response of mice irradiated in air. Filled symbols represent the response of hypoxic (clamped) tumours. Each data point represents the response of 5–86 mice.

development of chemosensitisation observed *in vitro* (Siemann, 1984). Until now no direct evidence for this requirement of hypoxia *in vivo* has been presented. Some indirect evidence exists, suggesting that hypoxia plays a role. Tumours with small hypoxic fractions show less enhanced damage to drugs than do the same tumours with larger hypoxic fractions. (Martin *et al.*, 1981; Sheldon & Batten, 1982; Spooner *et al.*, 1982). Tumours that lack radiobiologically hypoxic cells show no chemopotentiation unless they are made artifically hypoxic between MISO injection and treatment with BCNU (Wheeler *et al.*, 1984). However, the observed sensitisation exceeds what would be expected if the interaction between MISO and alkylating drugs is restricted to the small fraction of clonogenic and radiobiologically hypoxic tumour cells (Brown & Hirst, 1982; Hinchliffe *et al.*, 1983; Horsman *et al.*, 1984). So, while hypoxia appears to be necessary for the chemosensitisation to occur, significant effects can be seen in cells at oxygenation levels above what would render them radiobiologically hypoxic. This has been demonstrated by several investigators. Durand and Chaplin (1987) used the fluorochrome Hoechst 33342 to separate tumour cells in fractions as a function of distance from the blood vessels. They found that the chemosensitisation by MISO in KHT tumours treated wth CCNU was almost constant throughout the tumour, irrespective of the oxygenation status of the cells. *In vitro* the dependence of chemosensitisation by MISO on oxygen concentration has been examined (Mulcahy, 1984; Roizin-Towle *et al.*, 1986). It was found that the $K_m$ value, defined as the oxygen tension needed to generate half the maximum sensitisation was 400 p.p.m. Horsman *et al.* in a recently conducted study in EMT6 spheroids found constant chemosensitisation by MISO to melphalan as a function of depth within the spheroid. The binding of $^{14}C$-MISO, on the other hand, was found to increase with depth in the spheroid. Their data indicated that the majority of viable clonogenic spheroid cells (which were equally chemosensitised) were at oxygen tensions intermediate between those found in either aerobic or radiobiologically hypoxic cells (Horsman *et al.*, 1989). The observations from these *in vitro* studies are consistent with the *in situ* tumour data presented here, as some sensitisation of cells in the aerobic compartment seemed to occur. In our rather simplistic model this compartment contained all cells that were not radiobiologically hypoxic at the time of investigation.

In conclusion, this paper has presented *in situ* data on the chemosensitising effect of MISO on the response of solid tumour cells to CTX. Using a clamped tumour control assay it was found that the addition of MISO to CTX treatment caused a decrease in aerobic cell survival, which was 10-fold more than what was expected on an additive basis. In contrast to what has been observed *in vitro*, no synergistic effect was found on radiobiologically hypoxic cells. The exact role of hypoxia for the development of chemosensitisation seems to be complex and requires additional research in the future.

### Appendix: statistical methods

This appendix contains the equations used for estimating biological parameters and the standard errors of these.

### *Biological assumptions*

Naturally and artifically hypoxic cells were assumed to have identical dose-survival curves (Howes, 1969), described by the multi-hit cell-survival model with identical extrapolation number, *n*, and slope of the log-linear part of the survival curve, $D_0$. $D_0$ was taken to be 3.2 Gy (Suit *et al.*, 1965). The two types of cells were assumed to have the same biological properties if they survived treatment, e.g. the same potential for producing a tumour recurrence.

### *Statistical assumptions*

Standard errors of parameter estimates were obtained using the propagation-of-error technique. The dose required to control 50% of the tumours under oxic, $TCD_{50}$air, or clamped, $TCD_{50}$clamp, conditions was estimated by fitting a logit dose–response curve to the observed data. $TCD_{50}$ was assumed to have a normal distribution with standard error of the estimate calculated from the variance-covariance matrix. This assumption was found to be reasonable by Bentzen *et al.* (1988) using Monte Carlo simulations of dose–response data sets similar to those of the present study. Approximate 95% confidence limits may be calculated as the parameter estimate ± 1.96 (s.e.).

Assuming the tumour control probability to be determined solely from the radiosensitivity of the hypoxic tumour clonogens, the number of hypoxic tumour clonogens, $N_h$, is estimated as:

$$N_h = \exp(TCD_{50}air/D_o)\cdot \ln(2)/3 \quad (1a)$$

The factor ln(2)/3 is exp(ln(ln(2)) – ln(*n*)) in the derivation by Suit *et al.* (1965), assuming the extrapolation number *n* in the multi-hit cell-survival model to be equal to 3.

The standard error of $N_h$ may be estimated by:

$$s.e.(N_h) = N_h \cdot 1/D_o \cdot s.e.(TCD_{50}air) \quad (1b)$$

The total number of cells, $N_t$, is estimated from the $TCD_{50}$ under clamped conditions, when all clonogens are supposed to be hypoxic:

$$N_t = \exp(TCD_{50}clamp/D_o)\cdot \ln(2)/3 \quad (2a)$$

with standard error

$$s.e.(N_t) = N_t \cdot 1/D_o \cdot s.e.(TCD_{50}clamp) \quad (2b)$$

Dividing $N_h$ with $N_t$ (eqns 1a and 2a) yields the following expression for the hypoxic fraction:

$$HF = \exp(TCD_{50}air/D_o)/\exp(TCD_{50}clamp/D_o) = \exp((TCD_{50}air - TCD_{50}clamp)/D_o) \quad (3a)$$

The standard error of HF is estimated by:

$$s.e.(HF) = HF \cdot 1/D_o \cdot [s.e.(TCD_{50}air)^2 + s.e.(TCD_{50}clamp)^2]^{1/2} \quad (3b)$$

The number of aerobic cells, $N_a$:

$$N_a = N_t - N_h \quad (4a)$$

and

$$s.e.(N_a) = [s.e.(N_t)^2 + s.e.(N_h)^2]^{1/2} \quad (4b)$$

Finally, the surviving fraction after drug treatment was calculated as:

$$SF = N_1/N_2 \quad (5a)$$

where $N_1$ and $N_2$ are the number of cells after irradiation plus drug treatment and after irradiation alone, respectively.

$$s.e.(SF) = SF\,[s.e.(N_1)^2/N_1^2 + s.e.(N_2)^2/N_2^2]^{1/2} \quad (5b)$$

Equations 1b to 5b should be considered approximative and will only be valid if the coefficient of variation, that is the ratio between the standard error and the parameter estimate, is small. Simulation studies showed that equations 1b and 2b would produce reasonable standard error estimates, typically within 10% of the empirical standard deviation, provided that the s.e. of the $TCD_{50}$ was <1.5 Gy with a $TCD_{50}$ at 50 Gy.

We wish to thank Ms I.M. Johansen and Ms L. Skytte for excellent technical assistance and Dr M.R. Horsman for valuable comments during the preparation of this paper. Cyclophosphamide was kindly supplied by Farmitalia, Carlo Erba. Misonidazole was obtained through Roche Ltd, Copenhagen, by courtesy of Rud Hammer Jensen, MSc. This study was supported by the Danish Cancer Society, grant no. 86-079, and presented at the Sixth International Conference on Chemical Modifiers of Cancer Treatment, Paris, 21–25 March 1988.

### References

BENTZEN, S.M., JUUL CHRISTENSEN, J., OVERGAARD, J. & OVERGAARD, M. (1987). Some methodological problems in estimating radiobiological parameters from clinical data. *Acta Oncol.*, **27**, 105.

BROWN, J.M. (1982). The mechanisms of cytotoxicity and chemosensitization by misonidazole and other nitroimidazoles. *Int. J. Radiat. Oncol. Biol. Phys.*, **8**, 675.

BROWN, J.M. & HIRST, D.G. (1982). Effect of clinical levels of misonidazole on the response of tumor and normal tissues in the mouse to alkylating agents. *Br. J. Cancer*, **45**, 700.

DURAND, R.E. & CHAPLIN, D.J. (1987). Chemosensitization by misonidazole in CCNU-treated spheroids and tumours. *Br. J. Cancer*, **56**, 103.

GRAU, C. & OVERGAARD, J. (1988). Effect of cancer chemotherapy on the hypoxic fraction in a solid tumour measured using a local tumor control assay. *Radiother. Oncol.*, **13**, 301.

GRAU, C., ZACHARIAE, C., MAO, H.-S. & OVERGAARD, J. (1990). The *in vivo* response of a C3H mammary carcinoma to treatment with misonidazole, cyclophosphamide and radiation. *Acta Oncol.* (in the press).

HINCHLIFFE, M., MCNALLY, N.J. & STRATFORD, M.R.L. (1983). The effect of radiosensitizers on the pharmacokinetics of melphalan and cyclophosphamide in the mouse. *Br. J. Cancer*, **48**, 375.

HORSMAN, M.R., EVANS, J.W. & BROWN, J.M. (1984). Enhancement of melphalan induced tumor cell killing by misonidazole: an interaction of competing mechanisms. *Br. J. Cancer*, **50**, 305.

HORSMAN, M.R., WOOD, P.J. & BROWN, J.M. (1989). Misonidazole chemosensitization of EMT6 spheriods to melphalan. *Radiother. Oncol.*, **15**, 103.

HOWES, A.E. (1969). An estimation of changes in the proportions and absolute numbers of hypoxic cells after irradiation of transplanted C3H mouse mammary tumours. *Br. J. Radiol.*, **42**, 441.

MARTIN, W.M.C., MCNALLY, N.J. & DERONDE, J. (1981). The potentiation of cyclophosphamide cytotoxicity by misonidazole. *Br. J. Cancer*, **43**, 756.

MOORE, B.A., PALCIC, B. & SKARSGAARD, L.D. (1976). Radiosensitizing and toxic effects of the 2-nitroimidazole Ro-07-0582 in hypoxic and mammalian cells. *Radiat. Res.*, **67**, 459.

MULCAHY, R.T. (1984). Effect of oxygen on misonidazole chemosensitization and cytotoxicity *in vitro*. *Cancer Res.*, **44**, 4409.

ROIZIN-TOWLE, L., HALL, E.J. & PIRRO, J.P. (1986). Oxygen dependence for chemosensitization by misonidazole. *Br. J. Cancer*, **54**, 919.

SHELDON, P.W. & BATTEN, E.L. (1982). Potentiation in vivo of melphalan activity by nitroimidazole compounds. *Int. J. Radiat. Oncol. Biol. Phys.*, **8**, 635.

SIEMANN, D.W. (1982). Potentiation of chemotherapy by hypoxic cell radiation sensitizers–a review. *Int. J. Radiat. Oncol. Biol. Phys.*, **8**, 1029.

SIEMANN, D.W. (1984). Modification of chemotherapy by nitroimidazoles. *Int. J. Radiat. Oncol. Biol. Phys.*, **10**, 1585.

SMITH, E., STRATFORD, I.J. & ADAMS, G.E. (1982). Enhancing effect of pre-treatment of cells with misonidazole in hypoxia on their response to melphalan in air. *Br. J. Cancer*, **46**, 117.

SPOONER, D., PEACOCK, J.H. & STEPHENS, T.C. (1982). Enhancement of cytotoxic drugs by misonidazole in lewis lung tumors of different sizes, and mouse bone marrow. *Int. J. Radiat. Oncol. Biol. Phys.*, **8**, 643.

SUIT, H.D., SHALEK, R.J. & WETTE, R. (1965). *Cellular Radiation Biology*, p. 514. Williams & Wilkins: Baltimore.

SUTHERLAND, R.M., KENG, P., CONROY, P.J., MCDERMOTT, D., BAREHAM, B.J. & PASSALACQUA, W. (1982). *In vitro* hypoxic cytotoxicity of nitroimidazoles: uptake and cell cycle phase specificity. *Int. J. Radiat. Oncol. Biol. Phys.*, **8**, 745.

WHEELER, K.T., WALLEN, C.A., WOLF, K.L. & SIEMANN, D.W. (1984). Hypoxic cells and *in situ* chemopotentiation of the nitrosureas by misonidazole. *Br. J. Cancer*, **49**, 787.